# Book Reviews

**Published:** 1892-06

**Authors:** 


### *BOOK REVIEWS.*

---

CANCER. By Daniel Lewis, M. D., New York. Physicians' Leisure Library Series. Geo. S. Davis, Detroit, Mich.

This little volume contains the essential features of the course of clinical lectures delivered by the author on the subject of "Cancer" at the New York Post Graduate Medical School. The matter of caustic applications in the treatment of cancer is fully discussed and the cases indicated in which they are suitable. The author emphasizes the importance of early diagnosis, followed by prompt and radical removal, and thinks that in these circumstances cancer may be placed among the curable diseases.

---

ESSENTIALS OF PHYSICS. By Fred. J. Brockway, M. D., Assistant Demonstrator of Anatomy in the College of Physicians and Surgeons, New York. W. B. Saunders, Philadelphia.

The work here presented has been compiled from the larger works on Physics to meet the essential needs of the student of medicine. The compiler has been assisted also by the excellent lectures of Dr. Chandler delivered at the College of Physicians and Surgeons. From these lectures much useful scientific matter has been gotten that one may look for in vain in the average text book on Physics. The author has succeeded very well with his purpose and has given to medical students a book not voluminous or exhaustive, but sufficiently so to contain the principal facts of medical physics which it is useful or important for him to know.

---

THE PHYSICIAN AS A BUSINESS MAN. By J. J. Taylor, M. D., Philadelphia. Published by *The Medical World*, Philadelphia.

It is trite, but true, that the physician is a poor business man. It seems that either from the peculiar character of his daily work, or the never ending demands upon his time, or some other cause, he fails too often to pursue that sound business policy which other men would adopt.

The present small volume is an attempt to point out some of the reasons for the above and to suggest remedies; or "how to obtain the best financial results in the practice of medicine." It presents some good business rules, and abounds in wise and practical suggestions in regard to collections, fees, accounts, expenses, etc. All physicians will find here much to interest them.

and the younger members of the profession especially, who have not been initiated with the rugged ways of the business world, will obtain valuable information in these pages.

---

PRINCIPLES OF BACTERIOLOGY. By A. C. Abbott, M. D., Assistant in the Laboratory of Hygiene, University of Pennsylvania, Philadelphia. Philadelphia, Lea Bros. & Co.

The field of bacteriology is being studiously cultivated now, and the literature of the subject is being developed fast. Books and monographs are coming almost daily from the press. In the great number of those that have appeared there has been none that could better supply the practical needs of the student or physician than the above. Dr. Abbott's work is excellent, and his style is as simple as his directions are clear. The introduction gives the history of the decline and fall of the absurd doctrine of spontaneous generation. The next few chapters define, describe and classify bacteria. The methods of isolating, cultivating and examining them are next presented. The principles of sterilization and disinfection are also discussed.

The second part of the book is taken up with the study of certain diseases in their bacteriological relations. These are septicæmia, tuberculosis, typhoid fever, anthrax, diphtheria, etc. The author accepts the bacillus diphtheriæ of Loffler as the cause of the disease.

Such is a brief mention of a portion of the contents. The book will form a helpful companion to the student in the laboratory and to the physician who wishes to learn the technique of bacteriological study.

---

THE REFRACTION OF THE EYE. By Gustavus Hartridge, F. R. C. S. P. Blankiston, Son & Co., Philadelphia.

The fifth edition of this work upon ocular refraction is now out, and as the editor will attest, it has become one of the most popular treatises upon this important subject now before the profession. Its popularity is largely due to its simplicity and the clear manner in which the author presents the subjects subjoined with clinical cases which always make a subject more impressive to the mind's eye. The work is by no means exhaustive and on some subjects a few of the very latest methods of diagnosis are not touched upon. The work is a most excellent one and we heartily recommend it to those laboring in the field of ophthalmology. C. D. R.

